# Nanofiltration for Advanced and Reliable Drinking Water Treatment: Experimental Evaluation of Hybrid Pretreatment Systems and Fouling Control

**DOI:** 10.3390/membranes16060191

**Published:** 2026-06-01

**Authors:** Fazolrahman Bahig, Alimova Kulyash Kabpasovna, Nikita V. Martyushev, Boris V. Malozyomov, Vladislav V. Kukartsev, Tatyana Aleksandrovna Panfilova, Alena A. Stupina, Yadviga Aleksandrovna Tynchenko

**Affiliations:** 1 Institute of Architecture and Civil Engineering Named After T. K. Bassenov, Satbayev University, Almaty 050013, Kazakhstan; f.bahig@satbayev.university (F.B.); k.alimova@satbayev.university (A.K.K.); 2 Department of Information Technology, Tomsk Polytechnic University, 634050 Tomsk, Russia; 3 Department of Electrotechnical Complexes, Novosibirsk State Technical University, 630073 Novosibirsk, Russia; borisnovel@mail.ru; 4 Department of Software Engineering, Siberian Federal University, 660041 Krasnoyarsk, Russia; vlad_saa_2000@mail.ru; 5 Artificial Intelligence Technology Scientific and Education Center, Bauman Moscow State Technical University, 105005 Moscow, Russia; 6 Department of Applied Computer Science, Russian State Agrarian University—Moscow Timiryazev Agricultural Academy, 127434 Moscow, Russia; stupina@rgau-msha.ru; 7 Department of Technological Machines and Equipment for the Oil and Gas Complex, Siberian Federal University, 660041 Krasnoyarsk, Russia; 8 Center for Continuing Education, Bauman Moscow State Technical University, 105005 Moscow, Russia; 9 Department of Digital Management Technologies, Siberian Federal University, 660041 Krasnoyarsk, Russia

**Keywords:** nanofiltration, drinking water treatment, membrane fouling, hybrid systems, pretreatment, slow sand filtration, water sustainability, selective separation

## Abstract

Safe drinking water production from compositionally variable surface sources requires treatment systems that combine effective contaminant removal with stable membrane operation. This study experimentally evaluated a hybrid treatment train consisting of slow sand or zeolite pretreatment followed by NF for surface water representative of South-East Kazakhstan. The results showed that pretreatment reduced turbidity, iron, and organic load before the membrane stage, thereby improving flux stability and decreasing fouling propensity. Among the tested pretreatment options, zeolite provided the most favorable feed conditions and extended stable membrane operation. These findings demonstrate that the practical performance of NF depends not only on membrane properties but also on effective upstream conditioning of the feed stream. Under the tested recovery conditions, the selected operating regime produced permeate of acceptable final quality, confirming that hybrid pretreatment–NF systems are a robust option for drinking-water treatment from challenging surface sources.

## 1. Introduction

Ensuring a reliable supply of safe drinking water from polluted and compositionally variable surface sources remains a major engineering challenge worldwide, particularly in arid, semi-arid, and water-stressed regions, including Central Asia, the Middle East, North Africa, and parts of South Asia [1,2]. Surface waters in these regions are often characterized by high turbidity, elevated concentrations of natural organic matter, iron, and other dissolved contaminants, which reduce the efficiency and operational reliability of conventional treatment technologies [3,4,5,6]. Under such conditions, there is a growing need for treatment trains capable of combining high removal efficiency with stable long-term operation.

Among pressure-driven membrane processes, nanofiltration (NF) has emerged as a promising option for drinking-water treatment because it effectively removes multivalent ions, natural organic matter, and a broad range of micropollutants while operating at lower pressures than reverse osmosis [7]. Typical NF membranes are generally characterized by effective pore sizes on the order of 0.5–2 nm and by a molecular weight cut-off (MWCO) commonly in the range of approximately 200–1000 Da, which places them between ultrafiltration and reverse osmosis in terms of selectivity [8]. Although membranes operating within this separation range had been investigated earlier, nanofiltration became established as a distinct and commercially relevant membrane class mainly in the second half of the 1980s with the emergence of more stable composite membranes [9]. This combination of selectivity and moderate energy demand makes NF particularly attractive for advanced water treatment. However, its broader practical implementation remains constrained by membrane fouling, which leads to flux decline, more frequent chemical cleaning, increased operating costs, and reduced membrane service life [10].

Most recent studies have addressed this limitation primarily through membrane-material design, including surface modification, nanocomposite selective layers, and hydrophilic or antimicrobial coatings [11]. Although these approaches are scientifically important, their practical effectiveness under real surface-water conditions depends strongly on the quality of the feed stream delivered to the membrane stage. In this respect, pretreatment is not merely an auxiliary step, but a key factor governing process stability, fouling intensity, and overall system reliability. Therefore, NF should be assessed not only from the standpoint of membrane properties, but also as part of an integrated treatment system in which pretreatment and membrane separation operate as interdependent elements [12,13]. Accordingly, sand and zeolite filtration are considered here not as alternatives to nanofiltration, but as pretreatment stages used to condition the feed water and to evaluate how upstream treatment affects NF operation.

Previous studies on hybrid pretreatment–NF systems have shown that upstream conditioning can improve membrane operation by reducing particulate, colloidal, and organic loading before the NF stage. However, much of the available literature has focused either on a single pretreatment option, on membrane-centered performance without direct comparison of alternative low-energy media, or on feed waters that differ from highly variable natural surface sources. In particular, direct side-by-side comparisons of slow sand and zeolite pretreatment before the same NF unit under identical pilot-scale operating conditions remain limited. As a result, the extent to which differences in pretreatment media translate into downstream NF stability, fouling tendency, and practical operating robustness has not been sufficiently clarified [14]. The present study addresses this gap by experimentally comparing two simple pretreatment media within the same treatment train and by linking pretreatment performance to the subsequent behavior of the NF stage [15].

Against this background, the present study experimentally investigates a hybrid treatment train combining slow sand or zeolite pretreatment with NF for challenging surface water typical of South-East Kazakhstan [16,17,18]. The study focuses on how the choice of pretreatment medium influences feed-water conditioning, membrane operating stability, fouling behavior, and overall treatment performance under representative pilot conditions [19,20]. In this way, the work is intended to clarify the practical value of simple low-energy pretreatment strategies for improving the robustness of integrated NF-based drinking-water treatment.

## 2. Materials and Methods

The experimental study consisted of two sequential stages: pretreatment of raw surface water using pilot-scale sand and zeolite filters operated in parallel, and nanofiltration of the selected pretreated stream using a laboratory spiral-wound NF unit. This section describes the experimental facilities, operating conditions, analytical methods, and experimental protocol.

### 2.1. Experimental Facilities and Materials

#### 2.1.1. Slow-Rate Pretreatment Filtration

The study was carried out using surface water typical for water sources in South-East Kazakhstan (Almaty). This region faces persistent challenges related to drinking-water scarcity and surface-water contamination. The source water used in the experiment was selected to represent the typical characteristics of such waters: high turbidity (about 115 NTU), increased content of iron (up to 0.15 mg/L) and organic substances (permanganate oxidizability ~6.6 mgO_2_/dm^3^), as well as the presence of suspended solids [21,22]. These conditions motivated the selection of a treatment scheme including an effective pretreatment stage.

**Figure 1:**
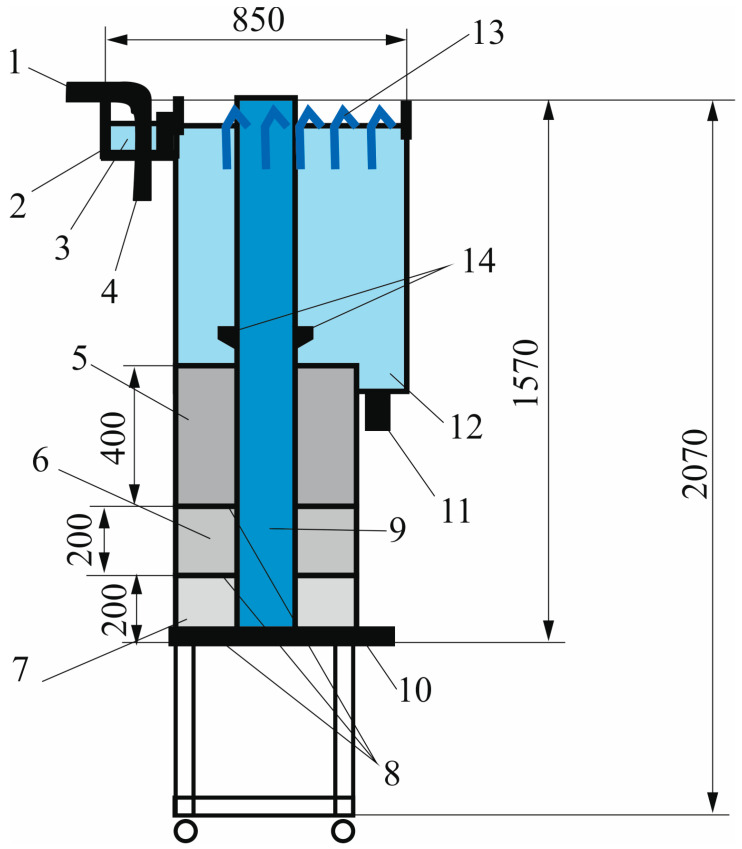
General view of the pilot slow-filter column used in the pretreatment stage. The numbered elements indicate the feed inlet, overflow and drain lines, layered filter bed, water-level inspection tubes, observation window, perforated outlet pipe, washout pocket, weir gutter, and regeneration lines.

For the pretreatment phase, two parallel pilot filter columns were used (Figure 1), identical in design but differing in filtration media. One column contained quartz sand and was operated as a slow sand filter (F–P), whereas the other contained natural zeolite and was operated as a slow-rate zeolite filter (F–C).

• Design: The filters were columns made of transparent plastic, equipped with a raw-water supply system, a drainage layer, a filter medium, an overflow device and pipes for the extraction of purified water and regeneration.
• Filter materials:
  ○ Sand filter (F–P): The filter bed consisted of three layers of quartz sand with a fraction of 0.8–2 mm (height 50 cm), 2–5 mm (10 cm) and 5–10 mm (10 cm). The lower drainage layer was made of gravel with a fraction of 10–20 mm (5 cm).
  ○ Zeolite filter (F–C): Natural zeolite with a fraction of 0.3–0.5 mm (50 cm) was used as the main load. Layers of zeolite with a fraction of 3 mm (5 cm), 5 mm (5 cm) and 5–10 mm (10 cm) were located above. The drainage layer consisted of gravel with a particle size of 5–10 mm (10 cm).
• Hydraulic loading regime: In both pretreatment lines, the filtration rate was maintained in the range of 0.1–0.2 m/h. In this manuscript, the term ‘slow filtration’ refers to this low hydraulic loading regime rather than to the type of filtering medium.

Both pilot filters were operated in downflow constant-head mode [23], with the water level maintained by the overflow device shown in Figure 1. The grain-size distribution of the filtering media is given above for each layer of the sand and zeolite beds. Regeneration of the zeolite filter was performed by hydraulic flushing through the dedicated regeneration lines shown in Figure 1, followed by restoration of the nominal filtration regime before the next experimental cycle.

**Figure 2:**
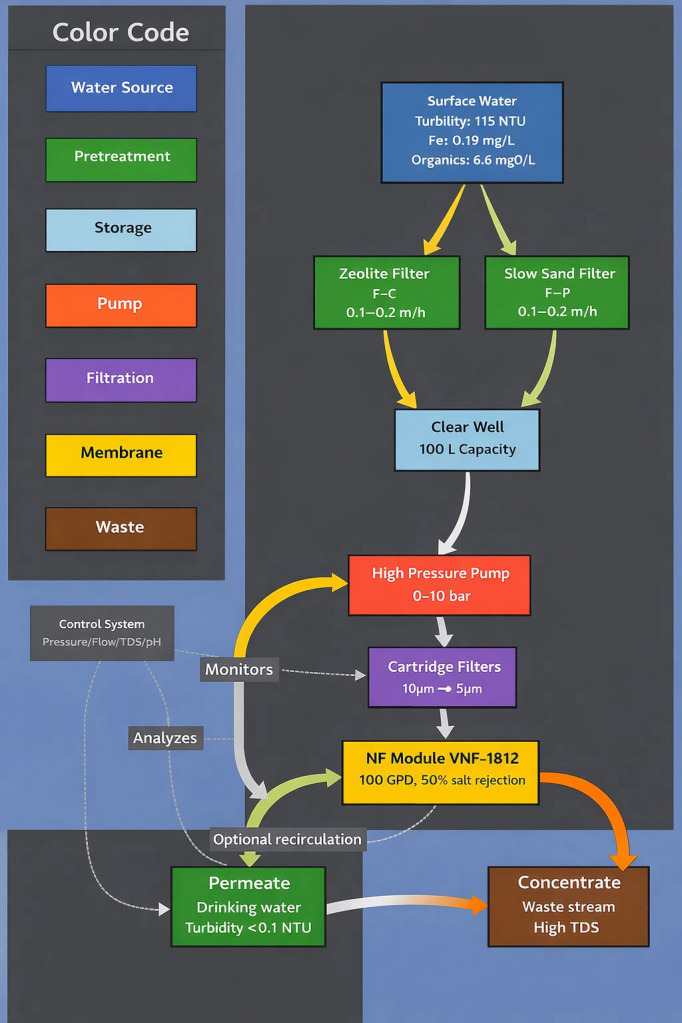
Simplified process flow diagram of the experimental pretreatment–nanofiltration system used in this study.

A simplified process flow diagram of the pilot treatment train is provided in Figure 2 to summarize the sequence of experimental stages. Raw surface water was first directed to the parallel sand and zeolite pretreatment filters operated at 0.1–0.2 m/h. After comparative evaluation of the two pretreatment options, the zeolite-filtered water, which showed the more favorable pretreatment performance [24], was selected for the downstream NF experiments and supplied to the nanofiltration unit through 10 and 5 μm cartridge filters.

The scheme shown in Figure 2 summarizes the sequence of the experimental treatment stages. After pretreatment in the parallel sand and zeolite filters, the clarified water was collected in the clear well and then supplied to the NF unit through a high-pressure pump and cartridge prefilters [25,26]. This configuration was used in all subsequent experiments on membrane performance and fouling behavior.

#### 2.1.2. Nanofiltration Unit

**Figure 3:**
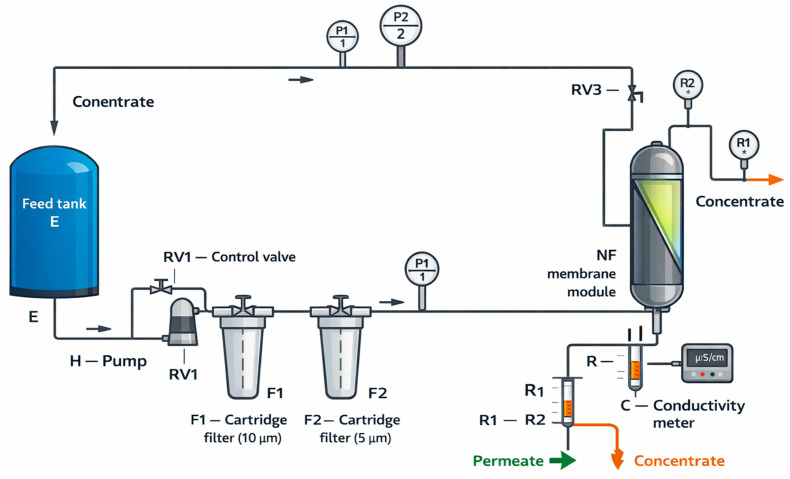
Flow scheme of the laboratory NF unit showing the feed tank, pump, control valves, cartridge prefilters, spiral-wound membrane module, pressure gauges, rotameters, and conductivity meter.

For tertiary treatment of the pretreated water, a laboratory NF unit was used (Figure 3). The feed solution was stored in tank E and delivered by pump H, while RV1 and RV2 were used to control pressure and flow rate. Cartridge filters F1 and F2 (10 and 5 μm) removed residual suspended matter before the feed entered the spiral-wound membrane module [27]. The module separated the stream into permeate and concentrate, which were discharged through separate lines and returned to tank E.

The P1 and P2 pressure gauges measure the pressure of the solution at the inlet and outlet of the membrane apparatus. The R1 and R2 rotameters are used to measure permeate and concentrate flow rates, respectively. The salinity of the streams was assessed using a conductivity meter (C) that measures the electrical conductivity of the solution.

**Figure 4:**
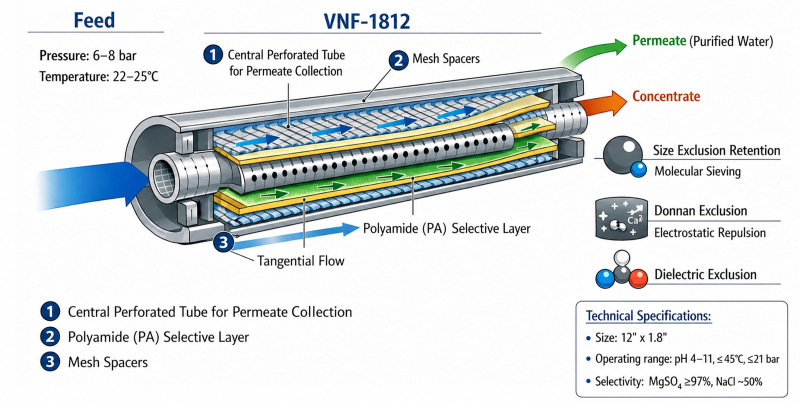
Schematic of the spiral-wound VNF-1812 module showing the feed, permeate, and concentrate flow paths.

Figure 4 shows the configuration of the spiral-wound nanofiltration module used in the laboratory unit.

The module used in the experiments was a spiral-wound VNF-1812 element comprising a polyamide thin-film composite membrane, a porous support layer, feed spacers, and a central perforated tube for permeate collection. During operation, the pretreated water entered the module at controlled pressure and temperature conditions and was separated in cross-flow mode into permeate and concentrate streams. The main technical characteristics of the NF unit are summarized below:

• Main element: 1812 standard size 1812 roll membrane element (length 12 in/298 mm, diameter 1.8 in/46 mm). A polyamide thin-film composite membrane (VNF-1812) was used with the following manufacturer-declared characteristics:
  - Nominal permeate capacity: 100 GPD (≈380 L/day).
  - Mean salt retention (by NaCl): 50%.
  - Mean hardness salt retention (for magnesium sulphate): ≥97%.
  - Maximum operating pressure: 21 bar (300 psi).
  - pH range for continuous operation: 4–11.

Equipment and fittings:

• Pump: A high-pressure centrifugal pump that allows continuous adjustment of the inlet pressure to the membrane module in the range of 0–10 bar.
• Pre-cleaning system: Two sequential 10 μm and 5 μm cartridge filters to remove suspensions and protect the membrane.
• Instrumentation: Pressure gauges at the inlet and outlet of the membrane module; rotameters for measuring permeate and concentrate flow rates; laboratory conductivity meter for online measurement of total salinity (salt content).
• Containers: Plastic tanks for initial solution, permeate and concentrate with a volume of 100 L.

### 2.2. Methods of Water Quality Analysis

Control of the treatment efficiency at each stage was carried out using standard physical and chemical methods [28].

• Spectrophotometry: A laboratory visible spectrophotometer Hach DR3900 (Hach Company, Loveland, CO, USA) (range 320–1100 nm) was used to determine the concentrations of key ions and indicators. The analysis was performed using TNTplus^®^ proprietary reagent kits using pre-defined calibrated methods for the determination of: permanganate oxidizability (PO), iron ions (Fe^2+^/Fe^3+^), sulphates (SO_4_^2−^), nitrates (NO_3_^−^), copper (Cu^2+^) and turbidity (in NTU).
• Electrochemical measurements: The pH value was measured with a laboratory pH meter with a combined electrode. Electrical conductivity (salt content) was measured with a portable conductivity meter.
• Gravimetric analysis: The mass concentration of suspended solids was determined by the standard method of filtering the sample through a pre-weighed membrane filter, followed by drying and weighing.

### 2.3. Experimental Protocol

The experimental protocol consisted of four consecutive stages. First, raw surface water was supplied to the sand and zeolite slow filters operating in parallel, and sampling was started after stabilization of the filtration regime (24–48 h). Second, samples of raw water and filtrates from both slow filters were collected twice daily (10:00 and 18:00) over a 30-day period and analyzed using the methods described above. Third, only the filtrate obtained from the zeolite filter was used as the feed stream for the nanofiltration unit operated in cross-flow mode at 6–7 bar and 22–25 °C, with monitoring of permeate flux and salt content. The sand-filtered water was not subjected to NF testing in the present study, because the objective of the second stage was to evaluate membrane behavior in an integrated treatment configuration based on the pretreatment option that showed the more favorable performance during the comparative pretreatment stage. Fourth, membrane fouling and cleaning behavior were evaluated by applying a chemical washing cycle whenever the normalized permeate flux declined by 10–15%; this threshold was intentionally selected as a conservative laboratory criterion to allow reproducible comparison of fouling progression and cleaning response before severe performance deterioration [29]. The cleaning procedure consisted of alkaline washing (NaOH, pH 12) followed by acid washing (citric acid, pH 2), after which flux recovery was determined.

### 2.4. Performance Metrics and Statistical Analysis

#### 2.4.1. Membrane-Process Parameters

Permeate flux (*J*): the volume of permeate (*V*) passing through a unit membrane area (*A*) per unit time (*t*). This parameter was used as the principal operational performance indicator. The standard process-performance equations used for permeate flux, rejection, and recovery were adopted from established membrane engineering methodology [30].

$$J=\frac{V}{At},\;\;\;l/\left(m^{2}\cdot h\right);$$

Rejection (*R*): The efficiency of the membrane in removing the target component. It is calculated in terms of the concentration of the component in the source water (*C_f_*) and in the permeate (*C_p_*).

$$R=1-\frac{C_{p}}{C_{f}}\cdot 100\%;$$

Recovery (*Y*): The ratio of the volume of permeate recovered (*V_p_*) to the volume of source water (*V_f_*), an important process parameter that affects salt formation.

$$Y=1-\frac{V_{p}}{V_{f}}\cdot 100\%.$$

#### 2.4.2. Pretreatment Removal Efficiency [31]

Pretreatment efficiency was evaluated as the percentage reduction in pollutant concentration relative to the raw water according to Equation (*X*):

*Removal efficiency* (%) = ((*C_raw_* − *C_filtrate_*)/*C_raw_*) × 100,

where *C_raw_* is the concentration of the pollutant in the raw water and *C_filtrate_* is the concentration of the pollutant in the filtrate.

#### 2.4.3. Statistical Analysis

**Figure 5:**
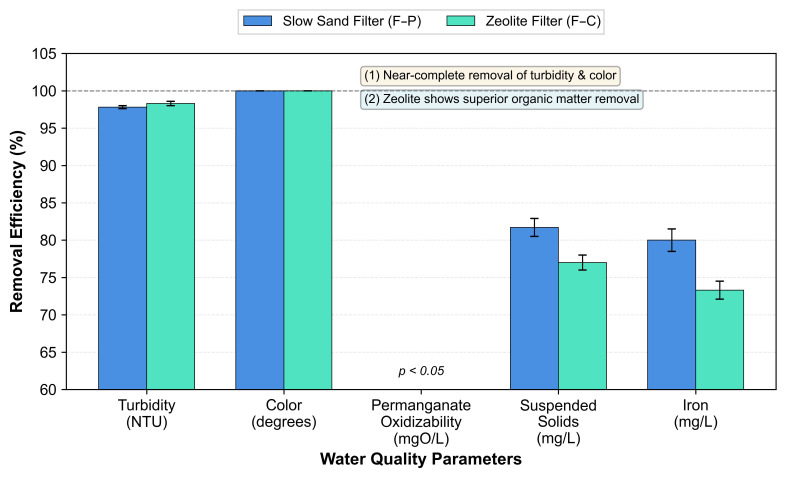
Comparative removal efficiency of key water quality parameters by slow sand (F–P) and zeolite (F–C) filters during the one-month pilot study. The figure is intended as a comparative summary of pretreatment performance over the monitoring campaign rather than a time-resolved presentation of daily fluctuations.

All analytical measurements were performed in triplicate for each collected sample, and the reported values are presented as arithmetic means ± standard deviations. During the 30-day monitoring campaign, raw-water and filtrate samples from each pretreatment line were collected twice daily, yielding 60 sampling points per parameter for each filter. For the comparative analysis of pretreatment performance shown in Figure 5, the removal-efficiency values for the sand and zeolite filters were compared using Student’s two-sided *t*-test at a significance level of *p* < 0.05. Prior to application of the parametric test, approximate normality of the data distributions and homogeneity of variances were assessed [32], and the assumptions were considered acceptable for the use of the *t*-test.

## 3. Results and Discussion

This section presents the results of a two-stage experiment on surface water purification. First, the effectiveness of two types of slow filters as a pre-treatment step is analyzed. Then, the operation of the nanofiltration membrane on the filtrate of the best of the filters was investigated, the effect of pre-treatment on the stability of the flow through the membrane was evaluated, and the parameters affecting the NF process were analyzed.

### 3.1. Pretreatment Performance of Slow Filters

The results of the 30-day monitoring campaign of water quality at the inlet and outlet of the sand (F–P) and zeolite (F–C) filters are summarized in Table 1. The reported values are given as averages with standard deviations based on the full sampling period and are intended to provide a comparative assessment of pretreatment performance [33]. The source water was characterized by increased turbidity, chromaticity, and iron content, which is typical for surface sources.

A histogram was constructed to visually compare the effectiveness of the two pretreatment filters and to better understand the quantitative data presented in Table 1 (Figure 5). It visualizes the average values of the removal efficiency of key water quality indicators for the entire monitoring period, with an indication of standard deviations. Asterisks indicate parameters for which the difference in efficiency between filters is statistically significant (*p* < 0.05, Student’s *t*-test). This graphical representation facilitates comparison of the two pretreatment media, highlights the principal differences in treatment performance, and supports selection of the preferred pretreatment option [34] for the downstream NF stage.

Data represent average removal percentages calculated from the monitoring period (see Table 1 for raw values). Error bars indicate standard deviation calculated from 60 sampling points per parameter for each pretreatment line collected during the 30-day monitoring campaign; each analytical determination at a given sampling point was performed in triplicate. Asterisks (*) denote statistically significant differences between the two-pretreatment media at *p* < 0.05 according to Student’s two-sided *t*-test. Key observations: Both filters achieved near-complete removal of turbidity and color. The zeolite filter demonstrated superior performance in reducing organic matter (permanganate oxidizability), attributed to its adsorption capacity. Both filters effectively removed suspended solids and iron, with the sand filter showing a slight, non-significant edge in iron removal. The zeolite filter’s ability to reduce organic load without altering ionic composition makes it the preferred pretreatment step for subsequent NF [35].

Figure 5 confirms that both pretreatment filters were highly effective in removing turbidity, color, suspended solids, and iron from the tested surface water. At the same time, the zeolite filter showed the most favorable overall pretreatment performance for subsequent nanofiltration because it provided a greater reduction in permanganate oxidizability, which is directly relevant to limiting membrane fouling by organic matter. This advantage is consistent with the sorption properties of zeolite and supports its selection as the preferred pretreatment medium for the downstream NF stage [36,37].

From an engineering perspective, the comparison between the two pretreatment options shows that both technologies are suitable for coarse clarification [38], but zeolite is more advantageous when the objective is not only particulate removal but also conditioning of the feed stream for a sensitive membrane process. Importantly, the zeolite filter achieved this improvement without materially altering the ionic composition of the water, which is beneficial for preserving stable NF operating conditions [39].

A dedicated analysis of short-term temporal fluctuations was beyond the scope of the present comparative study and will be considered in future work focused specifically on time-resolved pretreatment stability.

### 3.2. Nanofiltration Performance and Stability

In the second stage of the study, only the water pretreated by the zeolite filter (F–C) was fed to the nanofiltration unit. This choice was made because the zeolite filter showed the more favorable pretreatment performance in the comparative first stage, and the objective of the NF experiments was to evaluate membrane behavior under the integrated configuration selected as the most promising for downstream application. The main objectives at this stage were to evaluate the initial membrane performance [40,41], the dynamics of flux decline over time, and the response of the NF stage under the selected operating conditions used in this study.

#### 3.2.1. Initial Membrane Performance and Selectivity

Under the selected baseline operating conditions of this study, the NF stage showed stable initial performance and produced permeate that met the target drinking-water quality for the main monitored indicators [42,43]. To present these results more explicitly, the principal operating parameters and final permeate-quality characteristics are summarized in Table 2, including pressure, temperature, permeate recovery, flux, ion-rejection performance, and key water-quality indicators measured before and after the NF stage.

Table 2 consolidates the principal operating conditions of the NF stage together with the final permeate-quality characteristics achieved under the selected baseline regime. The data show that, at a transmembrane pressure of 6.0 ± 0.2 bar, temperature of 22 ± 1 °C, and permeate recovery of 15%, the membrane provided stable initial performance with a permeate flux of 10.5 ± 0.4 L·m^−2^·h^−1^. At the same time, the permeate quality was markedly improved relative to the zeolite-pretreated feed water: turbidity decreased to below 0.1 NTU, permanganate oxidizability to 0.8–1.2 mgO/dm^3^, and iron to below the detection limit. The NF stage also retained more than 93% of hardness ions and reduced the total dissolved solids content by approximately 50–60%, which is consistent with the characteristic selectivity of this membrane type.

#### 3.2.2. Flux Decline and Effect of Pretreatment

**Figure 6:**
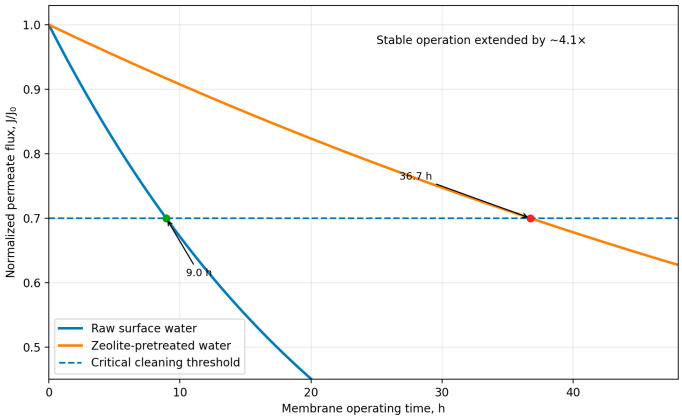
Comparative dynamics of normalized permeate flux (*J*/*J*_0_) during nanofiltration of raw surface water and zeolite-pretreated water, showing that zeolite pretreatment markedly slows flux decline by reducing permeate-productivity loss over the first 10–15 h from 35–40% to 10–12% and extending the operating interval before chemical cleaning by approximately 3–4 times.

The key result was the assessment of the stability of the membrane over time. Figure 6 shows the temporal evolution of normalized permeate flux during filtration of source water and water after the zeolite filter (F–C).

During nanofiltration of untreated raw water, a rapid (within 10–15 h) decrease in flow by 35–40% from the initial value was observed. This decline is attributed to rapid pore blocking and surface deposition by colloidal and organic matter [44].

Over the same period, the decrease was only 10–12%, and the time required to reach the conservative cleaning threshold used in this study was approximately 3–4 times longer than for untreated water.

To quantify this effect, Figure 6 shows the comparative dynamics of the normalized specific flux of permeate (*J*/*J*_0_) as a function of membrane operating time. The flux is normalized relative to its initial value (*J*_0_) for a visual comparison of the fouling rate.

The curves show that pretreatment substantially slows flux decline, reducing the loss of permeate productivity over the first 10–15 h from 35–40% to 10–12%. Under the conservative cleaning criterion adopted in this study (10–15% normalized flux decline), this corresponded to an approximately 3–4-fold extension of the operating interval before cleaning. Because industrial systems often use less conservative cleaning thresholds, the absolute cycle lengths reported here should not be interpreted as direct plant-scale values. However, the observed relative benefit of pretreatment remains relevant as evidence of improved membrane stability [45].

#### 3.2.3. Indirect Assessment of Membrane Fouling

The indirect evidence obtained from the cleaning response suggests the coexistence of several fouling components. The pronounced effect of acid cleaning is consistent with acid-soluble inorganic deposits, likely associated with hardness-related scaling near the membrane surface. The response to alkaline washing suggests the presence of organic foulants, including natural organic matter that reached the NF stage despite pretreatment. In turn, the longer filtration cycle after zeolite pretreatment indicates a reduced contribution of rapid colloidal pore blocking, although this interpretation was not verified by direct surface characterization.

#### 3.2.4. Pretreatment and Membrane Regeneration

These results lead to an important engineering conclusion. Zeolite pretreatment not only increases the interval between washes, but also changes the apparent fouling behavior toward a more reversible regime. Compared with operation without pretreatment, the membrane after zeolite prefiltration showed cleaning behavior more consistent with acid-removable inorganic scaling and a reduced contribution of rapid colloidal–organic fouling. However, this interpretation is indirect and is based on pretreatment performance and washing response rather than on direct compositional analysis of the fouling layer. This behavior may contribute to longer membrane service life by reducing the frequency and severity of chemical cleaning.

Taken together, these results indicate that zeolite pretreatment affects not only the rate of flux decline but also the apparent reversibility of membrane fouling under the selected operating and cleaning conditions. The longer operating interval before reaching the cleaning threshold, together with the observed response to alkaline and acid washing, suggests that upstream conditioning reduces the contribution of rapid colloidal–organic fouling and shifts the system toward a fouling regime that is operationally easier to manage. At the same time, because no direct physicochemical characterization of the deposited layer was performed, these mechanistic interpretations should be regarded as inferential rather than definitive. Therefore, this section is limited to interpretation of the observed process behavior and its engineering implications, whereas the formal conclusions of the study are presented only in Section 4.

## 4. Conclusions

This study experimentally demonstrated that nanofiltration performance for challenging surface water depends strongly on upstream pretreatment rather than on membrane properties alone. Both slow pretreatment filters provided effective clarification, but the zeolite filter produced the most favorable feed conditions for the downstream NF stage, especially because of its stronger reduction in organic load. Under the selected operating conditions, zeolite pretreatment substantially improved membrane stability by reducing the normalized flux decline over the first 10–15 h from 35–40% to 10–12% and by extending the operating interval before chemical cleaning by approximately 3–4 times.

The main novelty of the study lies in the experimental comparison of two simple low-energy pretreatment media within the same treatment train and in directly linking pretreatment performance to downstream NF stability and apparent fouling reversibility under identical pilot pretreatment conditions. In this way, the work supports a system-oriented interpretation of nanofiltration, in which feed-water conditioning is a decisive factor for practical process robustness.

From an engineering perspective, the results show that relatively simple pretreatment can improve the operational reliability of NF, reduce the frequency of cleaning interventions, and support the production of permeate of acceptable final quality under the tested regime. At the same time, the conclusions regarding fouling mechanisms remain indirect, since no direct physicochemical characterization of the fouling layer was performed, and the NF stage was tested only with zeolite-pretreated water. Therefore, further research should focus on direct foulant characterization, long-term pilot validation, and comparison of multiple NF membranes and pretreatment configurations under plant-relevant conditions.

## Figures and Tables

**Table 1:** Comparative efficiency of water purification with slow sand and zeolite filters (averaged data with standard deviations for the 30-day monitoring period).

Parameter	Unit	Raw Water	Sand Filter (F–P)	Removal Efficiency, %	Zeolite Filter (F–C)	Removal Efficiency, %
Turbidity	NTU	115 ± 12	2.5 ± 0.4	97.8	2.0 ± 0.3	98.3
Chromaticity	Hazen	25 ± 3	0	100	0	~100
Permanganate oxidizability	mgO/dm^3^	6.64 ± 0.5	3.22 ± 0.3	51.5	3.08 ± 0.2	53.6
Suspended solids	mg/dm^3^	0.213 ± 0.03	0.039 ± 0.01	81.7	0.049 ± 0.01	77.0
Iron (Fe, total)	mg/dm^3^	0.15 ± 0.02	0.03 ± 0.005	80.0	0.04 ± 0.006	73.3
Sulfates (SO_4_^2−^)	mg/dm^3^	22 ± 2	32 ± 3	n/a	23 ± 2	n/a
pH	-	7.16 ± 0.1	7.6 ± 0.1	n/a	7.23 ± 0.1	n/a

Note: For chromaticity, the outlet value was reported as 0 Hazen in both filters; therefore, the calculated removal efficiency is 100.0%. For sulfates and pH, removal efficiency is not applicable.

**Table 2:** Operating parameters and final permeate-quality characteristics of the NF stage under the selected baseline conditions.

Parameter	Unit	Feed to NF (Zeolite Filtrate)	Permeate	Rejection/Reduction, %
Pressure	bar	6.0 ± 0.2	—	—
Temperature	°C	22 ± 1	—	—
Permeate recovery	%	—	15	—
Permeate flux	L·m^−2^·h^−1^	—	10.5 ± 0.4	—
Conductivity	µS/cm	≈430 ± 25	≈205 ± 15	≈52
Total dissolved solids	% reduction or mg/L	280 ± 25 mg/L	115 ± 18 mg/L	50–60
Hardness ions (Ca^2+^ + Mg^2+^)	% rejection	—	—	>93
Turbidity	NTU	2.0 ± 0.3	<0.1	>95
Permanganate oxidizability	mgO/dm^3^	3.08 ± 0.2	0.8–1.2	61–74
Iron (total)	mg/dm^3^	0.04 ± 0.006	below detection limit	qualitative near-complete removal

## Data Availability

The original contributions presented in this study are included in the article. Further inquiries can be directed to the corresponding author.
